# Milk Intake, Sun Exposure, and Caffeinated Energy Drink Consumption in Children and Adolescents: Evidence, Uncertainty, and Implications for Peak Bone Mass Accrual

**DOI:** 10.3390/nu18132156

**Published:** 2026-07-03

**Authors:** Giorgos K. Sakkas, Ilias Ntoumas, Antonis Tsagkalis, Christina Karatzaferi

**Affiliations:** 1 Department of Physical Education and Sport Science, University of Thessaly, GR42100 Thessaly, Greece; intoumas@uth.gr (I.N.); atsagalis@gmail.com (A.T.); ck@uth.gr (C.K.); 2 Department of Life Sciences, School of Life and Health Sciences, University of Nicosia, 2417 Nicosia, Cyprus

**Keywords:** milk, dairy, calcium, vitamin D, sun exposure, caffeine, energy drinks, children, adolescents, peak bone mass, bone mineral density, DXA, Z-score, physical activity, sleep, GRADE

## Abstract

Background/Objectives: Childhood and adolescence are critical periods for bone mineral accrual and future skeletal reserve. Milk intake, sun exposure and caffeinated energy drink consumption are familiar lifestyle concepts, but they differ substantially in biological proximity and evidential strength. This structured narrative review critically evaluates these exposures in relation to peak bone mass accrual in youth. Methods: PubMed/MEDLINE, Scopus, Web of Science, the Cochrane Library and Google Scholar were searched from database inception to 23 June 2026. Search terms combined pediatric population terms with bone outcomes and exposure terms related to milk/dairy, calcium, vitamin D, sun exposure, physical activity, sleep, caffeine and energy drinks. A literature collection flowchart and a GRADE-informed evidence appraisal table are provided to improve transparency and clinical interpretability. Results: Evidence is strongest for adequate calcium intake, calcium-rich foods and weight-bearing physical activity as modifiable contributors to skeletal accrual. Vitamin D is essential for mineral homeostasis, but supplementation effects on bone density in otherwise healthy children are context-dependent and appear most relevant for deficiency prevention or treatment. Milk intake is best interpreted as a practical marker of calcium-rich dietary patterns rather than as the only route to calcium adequacy. Sun exposure is an indirect determinant of vitamin D status and is modified by season, latitude, skin pigmentation, clothing, sunscreen, adiposity and outdoor behavior. Direct evidence linking caffeinated energy drinks to impaired pediatric bone accrual is very limited. The relevance of caffeinated energy drink intake is better framed as indirect and hypothesis-generating, through possible displacement of calcium-rich beverages, sleep disruption and clustering with poorer lifestyle patterns. Conclusions: A prevention framework for pediatric bone health should emphasize calcium adequacy, avoidance of vitamin D deficiency, mechanical loading and correct pediatric DXA interpretation using Z-scores. Energy drinks can be included as a lifestyle concern, but conclusions should remain cautious because direct skeletal evidence is limited.

## 1. Introduction

Low bone mineral density and fragility fractures are usually recognized in adulthood, but the biological reserve that protects the skeleton is accumulated much earlier. Childhood and adolescence are characterized by rapid longitudinal growth, pubertal maturation, changes in body composition, and site-specific gains in bone mineral content (BMC), areal bone mineral density (aBMD), bone geometry and strength [1,2,3,4,5,6]. Peak bone mass is therefore a life-course target: lower-than-expected accrual during growth may leave a smaller skeletal reserve before the onset of adult bone loss [1,2,3,4,5,6].

Milk intake, sun exposure and caffeinated energy drink consumption are attractive public-health concepts because they are familiar to families and schools. However, they are not equivalent in evidential strength or biological proximity to bone outcomes. Milk is best considered as a practical dietary marker of calcium-rich food intake, high-quality protein and other nutrients; sun exposure is a determinant of cutaneous vitamin D synthesis but does not map directly onto serum 25-hydroxyvitamin D [25(OH)D]; and energy drinks represent a complex beverage category in which caffeine, sugar, stimulants, sleep disruption and displacement of healthier beverages may coexist [7,8,9,10].

The aim of this structured narrative review is to synthesize and critically appraise evidence linking milk/calcium intake, sun exposure/vitamin D status, sleep-related pathways and caffeinated energy drink consumption with skeletal accrual in children and adolescents. A secondary aim is to clarify pediatric densitometry terminology, because the relevant outcome is not an adult T-score diagnosis before 30 years of age, but rather suboptimal bone accrual and low BMC/aBMD Z-scores relative to age, sex and body size.

## 2. Methods: Structured Narrative Review Approach

This article is a structured narrative review informed by SANRA principles for narrative reviews [11]. It was not designed as a systematic review or meta-analysis; therefore, no pooled effect estimates were generated, and no formal risk-of-bias scoring was conducted for individual studies. To improve transparency, the literature search, eligibility framework, selection steps and synthesis priorities are described below and summarized in Table 1 and Figure 2.

PubMed/MEDLINE, Scopus, Web of Science, the Cochrane Library and Google Scholar were searched from database inception to 23 June 2026. The search strategy combined three concept blocks: pediatric population terms, bone outcome terms and lifestyle exposure terms. Example searches included: (child* OR adolescen* OR youth OR pediatric OR paediatric) AND (peak bone mass OR bone mineral content OR bone mineral density OR aBMD OR BMC OR DXA OR Z-score OR fracture) AND (milk OR dairy OR calcium OR vitamin D OR 25-hydroxyvitamin D OR sun exposure OR physical activity OR exercise OR sleep OR caffeine OR energy drink*). Searches were adapted to each database, and reference lists of key guidelines, position statements and systematic reviews were hand-searched to identify additional relevant sources.

Eligible sources included peer-reviewed systematic reviews, meta-analyses, randomized or controlled trials, prospective cohort studies, large cross-sectional studies, clinical guidance documents and position statements relevant to bone accrual in children and adolescents. Adult-only studies were excluded unless they informed densitometry terminology, caffeine safety thresholds or mechanisms that are frequently extrapolated to youth. Studies focused exclusively on severe chronic disease were not used as primary evidence for healthy pediatric populations, although they informed the discussion of measurement and clinical interpretation.

Because this review synthesizes lifestyle factors with different study designs and varying biological proximity to bone outcomes, the evidence was summarized using a GRADE-informed narrative framework. The GRADE approach rates certainty in a body of evidence as high, moderate, low or very low, with downgrading for limitations such as indirectness, inconsistency and imprecision [12]. In the present manuscript, this framework was used for interpretative grading only; it should not be interpreted as a full formal GRADE evidence profile with outcome-specific pooled estimates.

**Figure 1:**
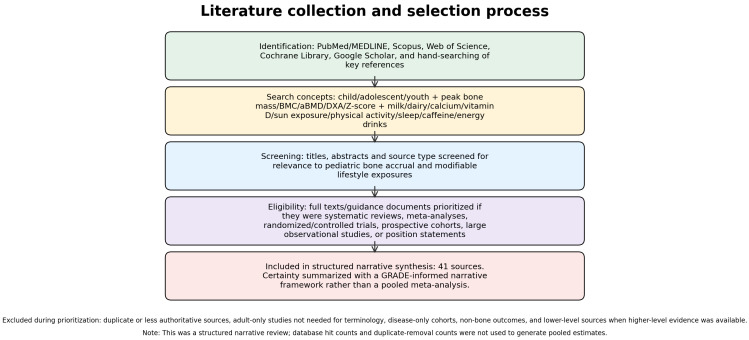
Literature collection and selection process for the structured narrative review. The figure documents identification, search concepts, screening, eligibility and inclusion steps, while acknowledging that the review was not a formal systematic review or meta-analysis.

Figure 1 provides the step-by-step literature collection and selection process used to structure the review. Because the review was narrative rather than systematic, database hit counts and duplicate-removal counts were not used to calculate pooled estimates; the flowchart instead documents the reproducible stages and the final included source set.

## 3. Conceptual Clarification: Milk, Sun and Energy Drinks Are Not Equivalent Exposures

A major interpretative issue is that the title-level exposures differ in how directly they relate to skeletal biology. Milk intake may be a source of calcium, protein, phosphorus, potassium and, in fortified products, vitamin D. Nevertheless, milk is not the only route to calcium adequacy, and the review should not be interpreted as arguing that dairy is indispensable for all children. Fortified plant-based beverages, calcium-set tofu, selected fish with edible bones and other foods can contribute to calcium intake when they are culturally acceptable and nutritionally adequate [13,14,15,16,17].

Sun exposure is likewise a proxy rather than a nutrient. Cutaneous vitamin D synthesis depends on season, latitude, time outdoors, skin pigmentation, clothing, sunscreen, air pollution and adiposity [14,16]. Therefore, residence in a sunny region cannot be assumed to guarantee vitamin D sufficiency. The clinically relevant biomarker is serum 25(OH)D, interpreted in context rather than as a simple reflection of sunlight availability [16].

**Figure 2:**
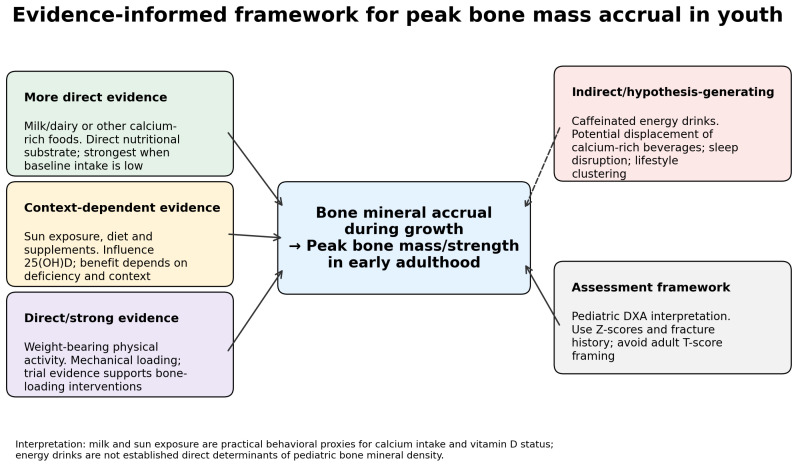
Evidence-informed framework linking milk/calcium intake, sun exposure/vitamin D status, weight-bearing physical activity, caffeinated energy drinks and pediatric DXA interpretation with bone mineral accrual in youth. The energy drink pathway is shown with a dashed arrow to indicate an indirect and hypothesis-generating relationship rather than established causality.

Energy drinks require the greatest caution. Unlike calcium intake, vitamin D status and weight-bearing physical activity, energy drink consumption has not been established as a direct determinant of pediatric bone mass accrual. Its relevance is mainly indirect: these beverages may displace milk or other calcium-rich options, cluster with poorer dietary patterns, contribute to high caffeine intake, and impair sleep. These pathways are plausible but should be treated as hypotheses or risk markers until prospective pediatric bone studies are available [7,8,9,10]. Figure 2 presents this evidence-informed framework and deliberately uses a dashed arrow for the energy drink pathway to indicate an indirect and hypothesis-generating relationship.

## 4. Peak Bone Mass Accrual During Growth

Peak bone mass reflects the maximum skeletal mass and strength achieved after growth and consolidation, although the timing varies by skeletal site and measurement method [1,2,3,4,5,6]. Adolescence is especially important because a substantial proportion of adult bone mass is accrued around the pubertal growth spurt [1,3,4]. This period is also when behavior patterns related to diet, physical activity, sunlight exposure, sleep and beverage choices become more independent from parental control.

The National Osteoporosis Foundation position statement concluded that lifestyle choices influence a meaningful proportion of adult peak bone mass and that calcium intake and physical activity are among the strongest modifiable evidence bases [4]. The same statement also emphasized that the evidence differs substantially across exposures, which is central to the present review: established determinants should not be blended with speculative exposures as if the evidence were equivalent [4].

Bone accrual should not be reduced to a single DXA value. BMC, aBMD, bone area, geometry, volumetric density and muscle–bone interactions all contribute to skeletal strength. DXA remains the most common clinical and research tool, but pediatric interpretation requires adjustment for growth, sex, pubertal status and body size.

## 5. Milk Intake, Calcium Adequacy and Bone Outcomes

Calcium is an established nutrient for skeletal mineralization, and requirements increase during rapid growth. Current dietary reference intakes identify 1300 mg/day as the recommended intake for children and adolescents aged 9–18 years [13,14,15]. Observational data indicate that many adolescents do not meet these targets, emphasizing the public-health relevance of calcium-rich dietary patterns.

Intervention evidence is more nuanced than a simple “more calcium equals more bone” message. A BMJ meta-analysis of randomized trials in healthy children found little or no effect of calcium supplementation on the femoral neck or lumbar spine, with small effects on the total body and upper limb sites [18]. A more recent synthesis in younger populations suggests that calcium supplementation can improve some bone mass parameters, but the magnitude, skeletal site, baseline intake and timing relative to peak bone mass matter [19]. Therefore, the strongest interpretation is that calcium adequacy is necessary for optimal bone accrual, whereas routine supplementation of already adequate children is less clearly beneficial.

Dairy-specific evidence supports a cautious, food-based interpretation. A systematic review of controlled trials concluded that supplementing the usual diet with dairy products significantly increased BMC during childhood, while evidence for linear growth was inconclusive [20]. A 2023 meta-analysis of randomized trials in children aged 3–18 years similarly found small but significant improvements in bone health indices with dairy product supplementation [21]. These findings support milk and dairy as practical calcium-rich options, but not as the only acceptable strategy for achieving calcium adequacy. Table 2 summarizes the certainty of evidence for milk/calcium, vitamin D, physical activity, sleep and energy drinks using a GRADE-informed narrative framework.

## 6. Sun Exposure, Vitamin D Status and Bone Outcomes

Vitamin D supports calcium absorption and mineral homeostasis, and severe deficiency can impair mineralization. However, the evidence for supplementation in otherwise healthy children is not uniform. Earlier systematic review evidence found no statistically significant effects of vitamin D supplementation on total body BMC or hip BMD in healthy children as a broad population [22]. A 2023 individual participant data meta-analysis of randomized trials reported that clinically important benefits from one year of vitamin D supplementation in healthy children and adolescents were unlikely across studied baseline 25(OH)D ranges, although the findings may not apply to children with symptomatic deficiency or rickets [23].

This distinction is essential for the revised argument. Low vitamin D status is common and clinically relevant, but supplementation should be interpreted according to baseline status, dose, adherence, calcium intake and population risk. The evidence supports preventing and treating deficiency, not indiscriminate high-dose supplementation for bone accrual in all children.

European and Mediterranean data show why the issue remains important. In the HELENA study, low vitamin D status was common among European adolescents [24]. In a nationally representative Irish teenage sample, calcium intake below estimated requirements and low vitamin D intake were common [25]. In Greece, schoolchildren have shown substantial vitamin D insufficiency despite the country’s high sunlight availability, with effects of seasonality, sex, urbanization and behavior [26]. The interaction between vitamin D status, physical activity and bone mass reported in adolescent cohorts suggests that vitamin D should be interpreted within a broader lifestyle and developmental context [27,28].

## 7. Physical Activity as a Higher-Level Comparator for Modifiable Bone Evidence

Although the title focuses on milk, sun and energy drinks, physical activity is included because it provides a comparator for the strength of evidence expected for a modifiable skeletal determinant. Bone responds to mechanical loading, especially during growth. Controlled-trial reviews indicate that weight-bearing and impact activities can enhance bone mineral accrual in children and adolescents, although optimal dose, type of activity, pubertal timing and skeletal site remain variable [29,30].

A systematic review of pediatric exercise interventions reported greater annual increases in BMC and aBMD in children assigned to exercise, with benefits most evident in prepubertal children [30]. This evidence is stronger and more direct than that for energy drinks and should anchor any public-health framework. Calcium adequacy may also modify the skeletal response to loading, emphasizing that nutrition and mechanical loading should be viewed as complementary rather than competing determinants [4,29,30].

## 8. Sleep as an Indirect Pathway Relevant to Bone Accrual

Sleep was added to the evidence table because the reviewer correctly noted that it is clinically relevant when discussing caffeinated energy drinks. Direct evidence linking sleep to pediatric bone accrual is less mature than evidence for calcium and mechanical loading, but cohort data suggest that sleep duration and sleep quality may be associated with bone stiffness in children and adolescents [31].

The relationship is likely indirect and bidirectional. Poor sleep may reduce daytime physical activity, alter appetite and dietary choices, and cluster with high screen time and caffeinated beverage intake. Conversely, evening caffeine consumption may worsen sleep quality or delay sleep timing. Observational studies of adolescents have linked energy drink or beverage patterns with sleep outcomes, but these studies do not prove that sleep disturbance mediates a causal effect of energy drinks on bone accrual [32,33]. Therefore, sleep is best treated as a plausible modifier and broader health target rather than an established standalone determinant of pediatric BMD.

## 9. Caffeinated Energy Drinks: Indirect Risk Marker Rather than Established Bone Determinant

The revised manuscript deliberately reframes energy drinks as an emerging behavioral risk marker, not as a proven cause of impaired bone accrual. Pediatric societies have expressed concern about energy drinks because of stimulant content, cardiovascular symptoms, sleep disturbance and broader adolescent health implications [7,8,10]. EFSA proposed 3 mg/kg body weight/day as a safety level for habitual caffeine intake in children and adolescents, but this does not establish energy drinks as safe or appropriate in pediatric dietary patterns [9].

To make the caffeine threshold clinically interpretable, Table 3 compares typical caffeine and sugar contents of selected energy drinks and conventional beverages. The table shows that one 500 mL can of a common energy drink can exceed the EFSA 3 mg/kg/day threshold for a 40 kg adolescent, whereas one 250 mL can may approach the threshold in a smaller child. Sugar-containing energy drinks also provide a large sugar load, which has broader metabolic and dental-health relevance even when a direct skeletal effect is not established [34,35,36,37,38,39].

Direct bone evidence is weak. A 2023 observational and Mendelian randomization study did not support a clear causal relationship between caffeine intake and BMD in children and adolescents [34]. Mechanistic concerns about caffeine and calcium economy exist but are not sufficient to claim that energy drinks independently reduce pediatric bone mass [35]. Therefore, statements linking energy drinks to bone should use terms such as “may”, “plausible”, “indirect”, “hypothesis-generating” and “requires prospective confirmation”.

The most defensible pathways are behavioral. First, energy drinks may displace milk, fortified beverages or meals that provide calcium and other bone-relevant nutrients. Second, caffeine consumed later in the day may reduce sleep quantity or quality, and sleep disturbance may influence growth, recovery, physical activity and diet quality [31,32,33]. Third, energy drink use may cluster with broader lifestyle patterns such as high screen time, low physical activity, sugar intake and irregular meals. These pathways justify including energy drinks in a lifestyle medicine framework, but they should not be ranked alongside calcium, vitamin D and weight-bearing activity as established skeletal determinants.

## 10. Pediatric DXA and Terminology: Integrating Z-Scores into the Argument

The distinction between T-scores and Z-scores is not a peripheral technicality; it changes the central claim of the article. T-scores compare an individual with a young adult reference population and are primarily used in adult osteoporosis classification. In children and adolescents, DXA interpretation should use Z-scores, with attention to growth, pubertal status, body size and fracture history [40,41].

The International Society for Clinical Densitometry states that osteoporosis should not appear in pediatric DXA reports without a clinically significant fracture history, and that “low bone mineral mass or bone mineral density” is the preferred terminology when pediatric BMC or aBMD Z-scores are less than or equal to −2.0 [40]. Thus, the concern addressed in this review is not that children will show adult-defined osteoporotic T-scores before the age of 30. The concern is that suboptimal nutrition and lifestyle during growth may reduce the probability of achieving genetically and developmentally appropriate peak bone mass.

This terminology also affects intervention goals. The aim is to support normal bone accrual, prevent deficiency states, encourage mechanical loading, and identify children with clinically relevant fracture patterns or low Z-scores. It is not to label otherwise healthy adolescents with adult osteoporosis categories.

## 11. Evidence Synthesis and Clinical Interpretation

The evidence base supports a graded interpretation. Stronger evidence exists for calcium adequacy, dairy/calcium-rich foods in low-intake children, vitamin D deficiency prevention and weight-bearing activity. Evidence for sun exposure is indirect because sun exposure affects 25(OH)D but is modified by many environmental and behavioral factors. Evidence for sleep is indirect and mainly observational. Evidence for caffeinated energy drinks and bone accrual is very low and indirect, although avoidance remains justified on broader pediatric health grounds [7,8,9,10,31,32,33,34,35]. Table 4 translates this evidence-graded interpretation into practical messages for families, schools, clinicians and researchers.

Clinically, the message can be framed without overclaiming: children and adolescents should have access to calcium-rich foods or nutritionally appropriate alternatives, avoid vitamin D deficiency, participate in regular impact and muscle-strengthening activities, sleep adequately, and avoid energy drinks. This statement is stronger as a preventive lifestyle recommendation than as a claim that energy drinks have proven direct skeletal toxicity.

Schools and families are practical settings for action. Bone-friendly environments can provide water, milk or fortified alternatives, balanced meals, outdoor play opportunities, physical education with impact loading, and policies discouraging energy drink availability. In clinical settings, counseling should be individualized according to dietary pattern, cultural preferences, sunlight behavior, skin protection, growth and puberty, fracture history, and any chronic disease or medication exposure affecting bone.

## 12. Limitations

This review has limitations. It is a structured narrative review rather than a systematic review or meta-analysis; therefore, it does not provide pooled estimates, formal certainty ratings for individual outcomes or exhaustive PRISMA-style database hit counts. The topic spans nutrition, pediatrics, endocrinology, exercise science, sleep and beverage behavior, and the literature varies widely in exposure measurement and bone outcomes.

Milk intake was often discussed through calcium adequacy because many bone studies measure calcium intake rather than milk as a distinct exposure. Sun exposure was discussed through vitamin D status because serum 25(OH)D, not sunlight itself, is the biological marker used in most bone studies. Energy drink evidence was discussed mainly through indirect pathways because direct pediatric bone data are sparse. The GRADE-informed table was used to increase clinical interpretability, but it should be viewed as a narrative evidence appraisal rather than a formal guideline-grade evidence profile.

## 13. Research Priorities

Future studies should distinguish beverage type, caffeine dose, timing of intake, sugar content, sleep, dietary calcium, vitamin D status, physical activity, pubertal maturation and socioeconomic context. Longitudinal cohorts and intervention studies are needed to test whether energy drink use predicts BMC, aBMD Z-scores, bone geometry or fracture outcomes after adjustment for these confounders.

For milk and dairy, studies should compare nutritionally equivalent calcium-rich alternatives and identify which children benefit most according to baseline intake, pubertal stage and activity level. For vitamin D, further work should focus on deficient or high-risk children, clinically relevant outcomes and safe, feasible population strategies that balance sun exposure with skin protection. Across all domains, pediatric bone outcomes should include Z-scores, body-size adjustment, pubertal stage, fracture history and, where feasible, measures of bone geometry and strength.

## 14. Conclusions

Milk intake, sun exposure and caffeinated energy drink consumption are useful public-facing concepts, but they should not be interpreted as equally established determinants of pediatric bone health. The most robust preventive message remains that children and adolescents need adequate calcium intake, avoidance of vitamin D deficiency and regular weight-bearing physical activity to support bone mineral accrual during growth.

Milk and dairy products can be practical contributors to calcium adequacy, while fortified alternatives may also be appropriate. Sun exposure contributes to vitamin D synthesis, but serum 25(OH)D depends on multiple biological and environmental factors and should guide clinical interpretation when assessment is indicated. Caffeinated energy drinks should be discouraged in children and adolescents primarily because of broader health concerns and plausible indirect pathways, not because direct evidence currently proves impaired bone accrual. A cautious, evidence-graded framework is therefore more scientifically defensible than a causal narrative linking energy drinks directly to low pediatric BMD.

## Figures and Tables

**Table 1:** Detailed search strategy and eligibility framework for the structured narrative review.

Component	Description
Databases	PubMed/MEDLINE, Scopus, Web of Science, Cochrane Library and Google Scholar; reference lists of key reviews, guidelines and position statements were hand-searched.
Timeframe	Database inception to 23 June 2026.
Population block	Child, adolescen, youth, puberty, pediatric, paediatric.
Bone/outcome block	peak bone mass, bone mineral content, bone mineral density, aBMD, BMC, DXA, Z-score, fracture, bone accrual.
Exposure block	milk, dairy, calcium, vitamin D, 25-hydroxyvitamin D, sun exposure, physical activity, exercise, sleep, caffeine, energy drink.
Example combined strategy	(child OR adolescen OR youth) AND (peak bone mass OR bone mineral density OR DXA OR Z-score) AND (milk OR dairy OR calcium OR vitamin D OR sun exposure OR sleep OR caffeine OR energy drink).
Priority evidence	Systematic reviews, meta-analyses, randomized/controlled trials, prospective cohorts, large cross-sectional studies, clinical guidance and densitometry position statements.
Synthesis approach	Narrative synthesis with explicit distinction between direct evidence, context-dependent evidence and indirect/hypothesis-generating pathways; GRADE-informed certainty categories used for interpretative summary.

**Table 2:** GRADE-informed evidence appraisal for modifiable exposures relevant to pediatric bone accrual.

Exposure/Domain	Main Evidence Base	GRADE-Informed Certainty for Pediatric Bone Accrual	Clinical Interpretation
Calcium adequacy	Established biological role; dietary reference intakes; trials and meta-analyses show site-specific effects, greatest relevance when intake is low [13,14,15,16,17,18,19].	Moderate	Promote adequacy; avoid overclaiming routine supplementation in children who already meet requirements.
Milk/dairy or calcium-rich foods	Controlled trials and meta-analyses support benefits for BMC/BMD indices, but dairy is not the only calcium source [20,21].	Moderate	Useful practical strategy for many children; fortified alternatives may be appropriate when nutritionally comparable.
Vitamin D deficiency prevention/treatment	Vitamin D is essential for mineral homeostasis; benefits depend on baseline status, dose, calcium intake and deficiency risk [16,22,23].	Moderate for deficiency prevention; low for universal supplementation in replete children	Assess risk and treat deficiency; do not imply universal skeletal benefit from supplementation in all children.
Sun exposure as vitamin D proxy	Indirect determinant of 25(OH)D modified by latitude, season, skin pigmentation, clothing, sunscreen, adiposity and behavior [16,24,25,26,27,28].	Low to moderate	Discuss as contextual contributor to vitamin D status, not a stand-alone bone intervention.
Weight-bearing physical activity	Controlled-trial reviews support bone-loading effects during growth, with site and pubertal-stage variability [29,30].	Moderate	Central preventive recommendation alongside nutrition.
Sleep	Limited observational pediatric bone data; plausible links through growth, recovery, activity and dietary behavior [31,32,33].	Low	Treat as a broader health and possible modifying factor; prospective mediation studies are needed.
Caffeinated energy drinks	Direct pediatric bone evidence is weak; caffeine/BMD study does not support clear causality; mechanisms are behavioral and indirect [7,8,9,10,32,33,34,35].	Very low for direct bone effect	Frame as behavioral risk marker/hypothesis, not established cause of low pediatric BMD.

**Table 3:** Approximate caffeine and sugar contents of selected beverages and clinical interpretation relative to the EFSA 3 mg/kg/day caffeine reference level for youth.

Beverage Example	Typical Serving	Caffeine per Serving	Sugar per Serving	Interpretation for a 40 kg Adolescent
Energy drink (Red Bull)	250 mL can	80 mg	27 g	Approx. 2 mg/kg; two cans would exceed 3 mg/kg/day [9,36].
Energy drink (Monster Original)	500 mL can	160 mg	55 g	Approx. 4 mg/kg; one can exceeds 3 mg/kg/day [9,37].
Cola-style soft drink	330 mL can	Approx. 33 mg	Approx. 35 g	Approx. 0.8 mg/kg; lower caffeine than energy drinks but substantial sugar [38,39].
Brewed coffee, unsweetened	Approx. 240 mL cup	Approx. 80–100 mg; variable by preparation	0 g unless sugar added	Approx. 2–2.5 mg/kg; caffeine exposure varies widely [38].
Milk, plain	250 mL glass	0 mg	Approx. 12 g naturally occurring lactose	No caffeine; contributes calcium and protein [15,38].
100% orange juice	Approx. 250 mL cup	0 mg	Approx. 21 g naturally occurring sugars	No caffeine; sugar content remains relevant to total diet [38].

**Table 4:** Practical clinical and public-health messages after evidence grading.

Audience	Cautious Message	Implementation Example
Families	Support bone accrual through calcium-rich foods, safe outdoor activity, sleep and regular movement.	Provide milk or fortified alternatives and water; avoid keeping energy drinks at home.
Schools	The school environment can support both nutrition and mechanical loading.	Water access, calcium-rich meals/snacks, physical education with jumping/running games, energy drink restrictions.
Clinicians	Assess growth, puberty, diet, vitamin D risk and fracture history; avoid adult T-score language.	Use DXA Z-scores when indicated and interpret results in clinical context.
Researchers	Energy drink–bone links require prospective bone-specific data.	Measure caffeine dose, beverage displacement, calcium, 25(OH)D, sleep, activity and pubertal status.

## Data Availability

No new data were created or analyzed in this structured narrative review. Data sharing is not applicable to this article.

## Abbreviations

The following abbreviations are used in this manuscript: 25(OH)D, 25-hydroxyvitamin D; aBMD, areal bone mineral density; BMC, bone mineral content; BMD, bone mineral density; DXA, dual-energy X-ray absorptiometry; EFSA, European Food Safety Authority; GRADE, Grading of Recommendations Assessment, Development and Evaluation; ISCD, International Society for Clinical Densitometry; RCT, randomized controlled trial.
